# A step forward for understanding the morbidity burden in Guinea: a national descriptive study

**DOI:** 10.1186/1471-2458-11-436

**Published:** 2011-06-06

**Authors:** Keita Mamady, Guoqing Hu

**Affiliations:** 1 Department of Epidemiology and Health Statistics, School of Public Health, Central South University, 110 Xiangya Road, 410078 Changsha, China

## Abstract

**Background:**

Little evidence on the burden of disease has been reported about Guinea. This study was conducted to demonstrate the morbidity burden in Guinea and provide basic evidence for setting health priorities.

**Methods:**

A retrospective descriptive study was designed to present the morbidity burden of Guinea. Morbidity data were extracted from the National Health Statistics Report of Guinea of 2008. The data are collected based on a pyramid of facilities which includes two national hospitals (teaching hospitals), seven regional hospitals, 26 prefectural hospitals, 8 communal medical centers, 390 health centers, and 628 health posts. Morbidity rates were calculated to measure the burden of non-fatal diseases. The contributions of the 10 leading diseases were presented by sex and age group.

**Results:**

In 2008, a total of 3,936,599 cases occurred. The morbidity rate for males was higher than for females, 461 versus 332 per 1,000 population. Malaria, respiratory infections, diarrheal diseases, helminthiases, and malnutrition ranked in the first 5 places and accounted for 74% of the total burden, respectively having a rate of 148, 64, 33, 32, and 14 per 1,000 population. The elderly aged 65+ had the highest morbidity rate (611 per 1,000 population) followed by working-age population (458 per 1,000 population) and children (396 per 1,000 population) while the working-age population aged 25-64 contributed the largest part (39%) to total cases. The sex- and age-specific spectrum of morbidity burden showed a similar profile except for small variations.

**Conclusion:**

Guinea has its unique morbidity burden. The ten leading causes of morbidity burden, especially for malaria, respiratory infections, diarrheal diseases, helminthiases, and malnutrition, need to be prioritized in Guinea.

## Background

A consistent description of the burden of diseases and injuries, and the risk factors associated with them, is an important input to health decision-making and planning processes [1]. The Global Burden of Disease (GBD) Study that was initiated in the early 1990s [2-4] provides a comprehensive assessment of the disease burden for all regions of the world, and yields important evidence for priority setting in health policy and research, and for the development of cost-effective health interventions. Since the GBD study introduced the disability-adjusted life year (DALY) -- a single measure to quantify the burden of diseases, injuries and risk factors -- in some countries, more and more countries have begun to use the DALY to set health priorities and support health decision-making [5-8].

As one of the least developed countries in the world, the Republic of Guinea (usually called Guinea) is characterized by high levels of vulnerability, mortality and malnutrition, limited access to basic social services, loss of coping mechanisms, and galloping spread of HIV/AIDS [9]. According to the Human Development Report 2007/2008, Guinea ranks 160th out of 177 countries in the ranking of human development index [10]. A number of health indicators of Guinea are among the most worrisome in the West Africa subregion [11].

Evidence-based health decision-making is especially important for Guinea to efficiently allocate its extremely limited health resources. On the other hand, little scientific evidence about Guinea has been produced in support of health decision-making up to now. We found very few scientific reports about the population health of Guinea through MEDLINE and search engines (like GOOGLE and YAHOO), and no mention of the disease burden. The GBD study included Guinea in the group of low- and middle-income countries in Africa, and reported the disease burden of this group rather than of each nation [1]. An early study in 1996 by Jha et al [12] reported the burden of disease based on mortality data. However the disease burden of Guinea might have changed in the past 12 years, and mortality alone does not convey the total burden of disease.

In addition, Guinea has the same problem of knowledge translation as most developing countries [13,14]. Few health officials are able to make full use of data to support their decision-making due to various reasons. The objective of this present study is to provide quantitative estimations of the national burden of non-fatal diseases for Guinea.

## Methods

### Data

A retrospective descriptive study was designed to demonstrate the morbidity burden of Guinea. The number of cases for calculating morbidity rates came from the Guinean Annual Health Statistics report 2008. Guinean Annual Health Statistics reports are based on the national data collection system. The national data collection system is a pyramid of facilities, comprising two national hospitals (teaching hospitals), seven regional hospitals, 26 prefectural hospitals, 8 communal medical centers, 390 health centers, and 628 health posts [15]. The data for all cases are recorded by highly trained health workers (doctors and nurses). With the help from WHO and other partners of development, Guinea uses standard clinical and laboratory methods to diagnose the cause of diseases [16]. The national public heath laboratory of Guinea regularly inspects all the laboratories and offers up-to-date training to laboratory assistants at local levels, so as to improve the quality of the laboratory results [15].

Skilled statisticians collect and process the data; then, send them to the Department of Health Information at the Ministry of Public Health, where a team of scientific community and specialized health experts validate the data and produce the annual health statistics report [15]. The complex health conditions that cannot be dealt with at health centers and posts are immediately referred to the prefectural hospital (the communal medical center is only at the level of the capital city, Conakry). In each region, there is a medical officer who coordinates and inspects the provision of health services and other activities such as regular organization of an intensive educational campaign to improve the quality, accuracy and reliability of health data. Guinea's population was estimated to be 9,958,190 inhabitants in 2008, on the basis of estimates of the 1996 census [15].

### Analysis

We did not calculate the disability adjusted life years (DALYs) to measure the disease burden due to the absence of basic indicators, because Cooper et al [17] pointed out that GBD projections from sub-Saharan Africa should not be used until data are available which correspond with modeled estimates. Mortality rates were excluded from this study because of the unavailability of fatal data in the Guinean Annual Health Statistics report 2008.

Morbidity rates were used to measure the burden of non-fatal diseases, calculated as the number of cases divided by the population × 1,000. The relative contributions of diseases were presented by sex and age group separately, since obvious sex and age differences were reported in the global burden of disease study [6]. We divided age into five groups: 0-4 years, 5-14 years, 15-24 years, 25-64 years, and 65 years and over. The Guinean health statistics report provides the data for 52 common and prevalent diseases. The 10 leading causes of diseases were presented to highlight the most important sources of morbidity burden, because they already accounted for more than 80% of the total morbidity rate for each sex- and age-specific group.

## Results

A total of 3,936,599 cases occurred in 2008, of which 57% were males and 43% were females (Table 1). The morbidity rate for males was higher than for females, 461 versus 332/1,000 population. The old adults aged 65+ had the highest morbidity rate (611/1,000) followed by the working-age population aged 25-64 (458.2/1,000) and children (396.4/1,000); the working-age population contributed the largest part (39%) to total cases.

**Table 1:** Morbidity rate/1,000 population in Guinea, 2008

Variables	Number of cases	Population	Percentage of cases	Morbidity rate/1,000 population
Total	3936599	9958190	100%	395
Sex				
Male	2242328	4860649	57%	461
Female	1694271	5097541	43%	332
Age group				
0-4	701155	1768670	18%	396
5-14	981218	2767748	25%	355
15-24	451299	1641504	12%	275
25-64	1520625	3318445	39%	458
65+	282302	461823	7%	611

**Figure 1:**
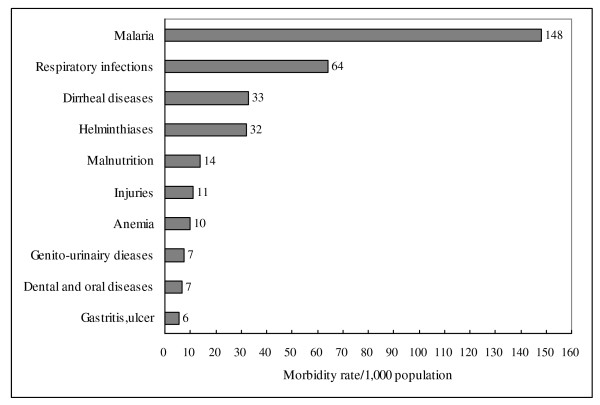
**Morbidity rate/1,000 population of the 10 leading cause of disease in Guinea, 2008**.

For the whole population, malaria, respiratory infections, diarrheal diseases, helminthiases, and malnutrition ranked in the first 5 places and accounted for 74% of the total burden, respectively having rates of 148, 64, 33, 32, and 14/1,000 population (Figure 1).

In males, malaria, respiratory infections were the most common diseases for all age groups (Table 2). Diarrheal disease, helminthiases, and malnutrition held the other top 5 places except for the age groups of 15-24 and 25-64, where injuries replaced malnutrition in the 5th place. The next 5 leading causes of diseases varied with age group. For instance, boys under 5 years experienced anemia, dermatologic diseases, ear-nose-throat infections, measles, and injuries. While for the elderly aged 65+, gastritis and ulcers, dermatologic diseases, injuries, hypertension, and articular diseases took the 6th to 10th places.

**Table 2:** Morbidity rates/1,000 population and percent of the 10 leading causes of diseases by age group in Guinea, (males, 2008)

Rank	0-4 years	5-14 years	15-24 years	25-64 years	65 years and over
1	Malaria	Malaria	Malaria	Malaria	Malaria
	174 (40%)	183 (45%)	98 (31%)	222 (39%)	146 (24%)
2	Respiratory infections	Respiratory infections	Respiratory infections	Respiratory infections	Respiratory infections
	104 (24%)	57 (14%)	55 (18%)	99 (17%)	98 (16%)
3	Diarrheal diseases	Helminthiases	Diarrheal diseases	Diarrheal diseases	Helminthiases
	39 (9%)	44 (11%)	23 (7%)	43 (7%)	67 (11%)
4	Helminthiases	Diarrheal diseases	Helminthiases	Helminthiases	Malnutrition
	34 (8%)	38 (9%)	22 (7%)	28 (5%)	36 (6%)
5	Malnutrition	Malnutrition	Injuries	Injuries	Diarrheal diseases
	16 (4%)	12 (3%)	18 (6%)	28 (5%)	29 (5%)
6	Anemia	Dental and Oral diseases	Malnutrition	Dental and Oral diseases	Gastritis, ulcer
	8 (2%)	10 (2%)	17 (6%)	17 (3%)	26 (4%)
7	Dermatologic diseases	Genito-urinairy diseases	Dermatologic diseases	Genito-urinairy diseases	Dermatologic diseases
	7 (2%)	9 (2%)	12 (4%)	14 (2%)	26 (4%)
8	E.N.T infections	Injuries	Dental and Oral diseases	Malnutrition	Injuries
	6 (1%)	8 (2%)	10 (3%)	13 (2%)	24 (4%)
9	Measles	Anemia	Gastritis, ulcers	Gastritis, ulcers	Hypertension
	6 (1%)	6 (2%)	8 (3%)	12 (2%)	23 (4%)
10	Injuries	Dermatologic diseases	Anemia	Dermatologic diseases	Articular diseases
	5 (1%)	6 (1%)	8 (3%)	12 (2%)	19 (3%)

Note: E.N.T infections mean ear, nose, and throat infections

In females, malaria was the first leading cause of diseases, as in males (Table 3). But for women/girls aged 5-14 and aged 25-64, diarrheal diseases took the second place. Compared with males, the most notable difference for women was that injuries were rarely found among the 10 leading causes except for aged 25-64 (the 5th place). Another important difference was that hypertension held the 10th place for women aged 15-64.

**Table 3:** Morbidity rates/1,000 population and percent of the 10 leading causes of diseases by age group in Guinea (females, 2008)

Rank	0-4 years	5-14 years	15-24 years	25-64 years	65 years and over
1	Malaria	Malaria	Malaria	Malaria	Malaria
	128 (36%)	120 (41%)	95 (40%)	124 (36%)	120 (22%)
2	Respiratory infections	Diarrheal diseases	Respiratory infections	Diarrheal diseases	Respiratory infections
	98 (27%)	35 (12%)	34 (14%)	38 (11%)	105 (19%)
3	Helminthiases	Respiratory infections	Helminthiases	Respiratory infections	Helminthiases
	35 (10%)	31 (11%)	23 (10%)	31 (9%)	58 (11%)
4	Diarrheal diseases	Helminthiases	Anemia	Helminthiases	Anemia
	28 (8%)	28 (9%)	13 (6%)	28 (8%)	34.55 (6%)
5	Malnutrition	Malnutrition	Malnutrition	Injuries	Malnutrition
	16 (4%)	11 (4%)	12 (5%)	14 (4%)	28 (5%)
6	Anemia	Anemia	Diarrheal diseases	Genitourinary diseases	Hypertension
	6 (2%)	10 (3%)	12 (5%)	14 (4%)	24 (4%)
7	E.N.T infections	Genitourinary diseases	Dental and Oral diseases	Malnutrition	Dermatologic diseases
	6 (2%)	9 (3%)	6 (2%)	13 (4%)	20 (4%)
8	Ocular infections	Dental and Oral diseases	Articular diseases	Anemia	Articular diseases
	6 (2%)	7 (2%)	4 (2%)	12 (3%)	18 (3%)
9	Measles	Ocular infections	Gastritis, Ulcers	Dental and Oral diseases	Gastritis, Ulcers
	6 (2%)	5 (2%)	4 (2%)	11 (3%)	17 (3%)
10	Dermatologic diseases	Dermatologic diseases	Hypertension	Hypertension	Dental and Oral diseases
	5 (1%)	4 (1%)	4 (2%)	7 (2%)	15 (3%)

Note: E.N.T infections mean ear, nose, and throat infections.

## Discussion

Our study is the first attempt to examine the non-fatal disease burden in Guinea. The morbidity rate for Guinea was 395/1,000 population in 2008; males had higher morbidity than females (461 vs. 332/1,000 population); all age groups were subject to diseases, with rates changing from 275 to 611/1,000 population; malaria, respiratory infections, diarrheal diseases and Helminthiases were the four leading causes of non-fatal diseases for the whole population, accounting for 70% of the morbidity burden. The order and morbidity rates of top 10 causes for sex- and age-specific groups are similar to those of the entire population except for a few differences.

The 10 leading causes of morbidity burden of Guinea are clearly different from those of the GBDS for the African region. Malaria, the first leading cause for Guinea, is absent from the three leading cause of disease for the African region based on DALYs although the respiratory infections and diarrheal diseases maintain the second and third positions in both studies [1]. Compared to country-based findings in Africa, our study presents findings similar to the report from sub-Saharan Africa [18] and dissimilar to the study from Tanzania [19] and South Africa [20]. In fact, there are no countries showing the same ten leading causes of diseases.

The agreement and disagreement between this study and the existing studies might be partly due to the differences in the measurement method and the year of research. On the other hand, they might strongly indicate that the burden of disease varies with nations. Würthwein et al [18] reported that the ranking of diseases in sub-Saharan Africa by the burden of disease differs substantially from the ranking in the GBDS, and suggested that local health policy should rather be based on the local burden of disease measurement instead of relying on extrapolations that might not represent the true burden of disease structure by cause.

Our findings characterize a unique spectrum of non-fatal disease burden in Guinea. On the one hand, infectious diseases are the most important threat for Guineans, such as malaria, respiratory infections, diarrheal diseases and helminthiases. The persistence of diarrhea illnesses might be due to unhygienic eating and drinking. This presence of measles as the ninth leading cause of morbidity in children under five years may reflect the low coverage of vaccination in Guinea [21].

On the other hand, non-communicable diseases and injuries are also common in Guinea as well as in other African countries [22,23]. These characteristics need to be considered when the government of Guinea prioritizes its health actions.

At the same time, we clearly realize that it is very difficult for Guinea to improve the current health situation without external efforts. Although few studies reveal the real problems in Guinea, Guinea also has serious problems similar to those in other under-developed countries, like the lack of trained health workforce [24-26], the shortage of high-quality research [27,28], and the low coverage of information technology [29]. No doubt, actions are needed to increase the help from developed countries to under-developed countries.

This study was limited by the completeness and the quality of data. First, the mortality data were excluded from this study due to the unavailability of data, preventing us from presenting a complete portrait of the disease burden. Second, the real morbidity burden might be underestimated because the national health statistics report does not include patients who go to private clinics or other non-governmental facilities or are treated at home. Third, many Guineans choose traditional healers rather than hospital care because of high cost and scarcity of hospital service; for this reason, the medical records of these patients are excluded from data collection, thus leading to the underestimation of morbidity rate [30]. Fourth, the classification of diseases of the national health statistics report and the lack of related parameters prevented us from calculating the burden of non-fatal diseases using the years lost due to disability.

## Conclusions

To conclude, the ten leading causes account for about 90% of total morbidity burden, and should be prioritized in Guinea. Actions are also needed to improve the health information system so as to provide accurate, complete, and timely evidence for decision-making.

## List of abbreviations

GBD: global burden of disease; DALY: the disability-adjusted life year.

## Competing interests

The authors declare that they have no competing interests.

## Authors' contributions

KM and GH conceived of the idea, completed data analysis and wrote the paper. Both authors read and approved the final manuscript.

## Pre-publication history

The pre-publication history for this paper can be accessed here:

http://www.biomedcentral.com/1471-2458/11/436/prepub
